# Honokiol activates AMP-activated protein kinase in breast cancer cells via an LKB1-dependent pathway and inhibits breast carcinogenesis

**DOI:** 10.1186/bcr3128

**Published:** 2012-02-21

**Authors:** Arumugam Nagalingam, Jack L Arbiser, Michael Y Bonner, Neeraj K Saxena, Dipali Sharma

**Affiliations:** 1 Department of Oncology, Johns Hopkins University School of Medicine and the Sidney Kimmel Comprehensive Cancer Center at Johns Hopkins, Baltimore MD 21231; 2 Department of Dermatology, Emory University School of Medicine, Winship Cancer Institute, Atlanta, GA 30322; 3 Department of Medicine, University of Maryland School of Medicine, Baltimore, MD 21201

## Abstract

**Introduction:**

Honokiol, a small-molecule polyphenol isolated from magnolia species, is widely known for its therapeutic potential as an antiinflammatory, antithrombosis, and antioxidant agent, and more recently, for its protective function in the pathogenesis of carcinogenesis. In the present study, we sought to examine the effectiveness of honokiol in inhibiting migration and invasion of breast cancer cells and to elucidate the underlying molecular mechanisms.

**Methods:**

Clonogenicity and three-dimensional colony-formation assays were used to examine breast cancer cell growth with honokiol treatment. The effect of honokiol on invasion and migration of breast cancer cells was evaluated by using Matrigel invasion, scratch-migration, spheroid-migration, and electric cell-substrate impedance sensing (ECIS)-based migration assays. Western blot and immunofluorescence analysis were used to examine activation of the liver kinase B1 (LKB1)-AMP-activated protein kinase (AMPK) axis. Isogenic LKB1-knockdown breast cancer cell line pairs were developed. Functional importance of AMPK activation and LKB1 overexpression in the biologic effects of honokiol was examined by using AMPK-null and AMPK-wild type (WT) immortalized mouse embryonic fibroblasts (MEFs) and isogenic LKB1-knockdown cell line pairs. Finally, mouse xenografts, immunohistochemical and Western blot analysis of tumors were used.

**Results:**

Analysis of the underlying molecular mechanisms revealed that honokiol treatment increases AMP-activated protein kinase (AMPK) phosphorylation and activity, as evidenced by increased phosphorylation of the downstream target of AMPK, acetyl-coenzyme A carboxylase (ACC) and inhibition of phosphorylation of p70S6kinase (pS6K) and eukaryotic translation initiation factor 4E binding protein 1 (4EBP1). By using AMPK-null and AMPK-WT (MEFs), we found that AMPK is required for honokiol-mediated modulation of pACC-pS6K. Intriguingly, we discovered that honokiol treatment increased the expression and cytoplasmic translocation of tumor-suppressor LKB1 in breast cancer cells. LKB1 knockdown inhibited honokiol-mediated activation of AMPK and, more important, inhibition of migration and invasion of breast cancer cells. Furthermore, honokiol treatment resulted in inhibition of breast tumorigenesis *in vivo*. Analysis of tumors showed significant increases in the levels of cytoplasmic LKB1 and phospho-AMPK in honokiol-treated tumors.

**Conclusions:**

Taken together, these data provide the first *in vitro *and *in vivo *evidence of the integral role of the LKB1-AMPK axis in honokiol-mediated inhibition of the invasion and migration of breast cancer cells. In conclusion, honokiol treatment could potentially be a rational therapeutic strategy for breast carcinoma.

## Introduction

Breast cancer is one of the most common cancers and the second leading cause of cancer-related mortality in women. About 226,870 new cases of invasive breast cancer and about 63,300 new cases of carcinoma *in situ *will be diagnosed in 2012, according to the latest estimates for breast cancer in the United States by American Cancer Society. Despite major advances in screening programs and development of various targeted therapeutic approaches, mortality related to breast cancer still remains at a staggering high level, with approximately 1 in 35 women dying of breast cancer. Available therapies, including radiation, endocrine, and conventional chemotherapy, are often limited by high toxicity, lower efficacy, therapeutic resistance, and therapy-related morbidity. Therefore, more-effective therapeutic strategies are clearly needed to combat breast cancer and to reduce morbidity and mortality.

The importance of active constitutive agents in natural products has become increasingly apparent, owing to their potential cancer preventive as well as therapeutic properties [1,2]. In traditional Asian medicine, root and stem bark of *Magnolia *species have been used for centuries to treat anxiety, nervous disorders, fever, gastrointestinal symptoms, and stroke [3]. Therapeutic benefits of *Magnolia *species have been attributed to honokiol, a natural phenolic compound isolated from an extract of seed cones from *Magnolia grandiflora *[3,4]. Honokiol has shown antithrombocytic, antibacterial, antiinflammatory, antioxidant, and anxiolytic effects, and it may prove beneficial against hepatotoxicity, neurotoxicity, thrombosis, and angiopathy [3]. Two pioneering studies showing the remarkable inhibitory effects of honokiol on mouse skin-tumor promotion and demonstrating efficacy of honokiol against established tumors in mice [5,6] ascertained the anticancer potential of honokiol. Subsequent studies showed the anticancer activities of honokiol in many cancer cell lines and tumor models [7-11].

Honokiol has been found to alter many cellular processes and to modulate molecular targets that are known to affect apoptosis, growth, and survival of tumor cells. A review of previous studies suggests that the mechanism by which honokiol causes growth arrest and cell death may be cell-line/tumor-type specific and involve many signaling pathways. For instance, Bax upregulation has been observed in some but not in other cellular systems [7,12]. Honokiol decreases phosphorylation of ERK, Akt, and c-Src to induce apoptosis effectively in SVR angiosarcoma cells [3], inhibits the ERK signaling pathway to exert antiangiogenesis activity [13], but activates ERK in cortical neurons to induce neurite outgrowth [14,15]. In chronic lymphocytic leukemia (CLL), honokiol causes apoptosis through activation of caspase 8, followed by caspase 9 and 3 activation [7]. Honokiol-mediated increased cleavage of Mcl-1 and downregulation of XIAP as well as BAD upregulation is observed in multiple myeloma, whereas Bid, p-Bad, Bak, Bax, Bcl-2, and Bcl-xL remain unchanged [12]. Honokiol also inhibits the NF-κB signaling pathway, thus affecting expression of many downstream genes in endothelial cells, human monocytes, lymphoma, embryonic kidney cells, promyelocytic leukemia, multiple myeloma, breast cancer, cervical cancer, and head and neck cancer [16-19]. Thus, honokiol elicits several cellular responses and modulates multiple facets of signal transduction.

In the present study, we specifically investigated the effect of honokiol on the malignant properties of breast cancer cells, including migration and invasion, and also examined the underlying molecular mechanisms. Intriguingly, we discovered that honokiol increases the expression of tumor-suppressor LKB1 to modulate the signaling pathway involving the AMPK-pS6K axis. We directly tested the requirement of AMPK and LKB1 in honokiol-mediated inhibition of malignant properties of breast cancer cells. Our results showed that LKB1 and AMPK are integral molecules required for honokiol-mediated modulation of 4EBP1-pS6K and inhibition of migration and invasion of breast cancer cells.

## Materials and methods

### Cell culture and reagents

The human breast cancer cell lines, MCF7 and MDA-MB-231, were obtained from the American Type Culture Collection and maintained in DMEM supplemented with 10% fetal bovine serum (FBS) (Gemini Bioproducts, Woodland, CA, USA) and 2 μ*M *L-glutamine (Invitrogen, Carlsbad, CA, USA). Cell-line authentication was done by analysis of known genetic markers or response (for example, expression of estrogen receptor and p53 and estrogen responsiveness) [20]. AMPK-null and AMPK-WT immortalized MEFs were kindly provided by Dr. Keith R. Laderoute (SRI International, Menlo Park, CA, USA) [21]. Honokiol is a natural product extracted from seed cone of *Magnolia grandiflora*, as previously described [6]. Antibodies for p-AMPK (phospho-AMPK), AMPK, ACC, p-ACC (phospho-ACC), pS6K, p-pS6K (phospho-S6K), 4EBP1, p-4EBP1 (phospho-4EBP1), p-Akt (phospho-Akt), Akt, and LKB1 (3047) were purchased from Cell Signaling Technology (Danvers, MA, USA).

### LKB1 stable knockdown using lentiviral short-hairpin RNA

Five pre-made lentiviral LKB1 short-hairpin RNA (shRNA) constructs and a negative control construct created in the same vector system (pLKO.1) were purchased from Open Biosystems (Huntsville, AL, USA). Paired LKB1 stable knockdown cells (MCF7 and MDA-MB-231) were generated by following our previously published protocol [22].

### Cell-viability assay

Cell-viability assay was performed by estimating the reduction of XTT (2,3-bis(2-methoxy-4-nitro-5-sulfophenyl)-2*H*-tetrazolium-5-carboxyanilide), by using a commercially available kit (Roche) [23]. Breast cancer cells were treated with honokiol as indicated.

### Clonogenicity assay

For colony-formation assay [24], MCF7 and MDA-MB-231 cells were treated with honokiol as indicated for 10 days; colonies containing > 50 normal-appearing cells were counted.

### Anchorage-independent soft-agar growth assay

Anchorage-independent growth of MCF7 and MDA-MB-231 cells in the presence of honokiol treatment was determined by colony formation on soft agar [25]. Colonies were counted in five randomly selected fields at ×10 magnification by using Olympus IX50 inverted microscope.

### Scratch-migration assay

Migration assay was performed according to our published protocol [26]. Cells were treated with honokiol as indicated. Plates were photographed after 24 and 48 hours at the identical location of the initial image.

### Electric cell-substrate impedance sensing *(*ECIS) wound-healing assay

Wound-healing assay was performed by using the ECIS (Applied BioPhysics, Troy, NY, USA) technology and following our previously established protocol [27].

### Spheroid migration assay

MDA-MB-231 and MCF7 cells (1.5 × 10^4^) were seeded in 0.5% agar-coated plates and cultured on an orbital shaker (100 rpm) for 48 hours in a humidified atmosphere containing 5% CO_2 _at 37°C. Intact tumor spheroids were selected and transferred to six-well plates. The spheroids were treated with honokiol, as indicated. After 48 hours of incubation, spheroids were fixed with 10% buffered formalin in PBS and stained with crystal violet. The migration of cells from spheroids was observed under a light microscope.

### Invasion assay

For an *in vitro *model system of metastasis, Matrigel invasion assay [28] was performed by using a Matrigel invasion chamber from BD Biocoat Cellware (San Jose, CA, USA). The slides were coded to prevent counting bias, and the number of invaded cells on representative sections of each membrane were counted under light microscope. The number of invaded cells for each experimental sample represents the average of triplicate wells.

### Western blotting

Whole cell lysate [23] was prepared by scraping MCF7 and MDA-MB-231 cells in 250 μl of ice-cold modified RIPA buffer. An equal amount of protein was resolved on sodium dodecylsulfate polyacrylamide gel, transferred to nitrocellulose membrane, and Western blot analysis was performed. Immunodetection was performed by using enhanced chemiluminescence (ECL system; Amersham Pharmacia Biotech, Arlington Heights, IL, USA) according to manufacturer's instructions.

### Immunoprecipitation assay

Immunoprecipitation of LKB1 was performed by following the previously published protocol [23] by using anti-LKB1 antibody followed by immunoblotting with anti-STRAD antibody.

### Immunofluorescence and confocal imaging

Breast cancer cells (5 × 10^5 ^cells/well) were plated in four-well chamber slides (Nunc, Rochester, NY, USA) followed by treatment with honokiol and subjected to immunofluorescence analysis as described [22]. Fixed and immunofluorescently stained cells were imaged by using a Zeiss LSM510 Meta (Zeiss) laser scanning confocal system configured to a Zeiss Axioplan 2 upright microscope with a 63XO (NA 1.4) plan-apochromat objective. All experiments were performed multiple times by using independent biologic replicates.

### Breast tumorigenesis assay

MDA-MB-231 (5 × 10^6^) cells in 0.1 ml of HBSS were injected subcutaneously into the right gluteal region of 4- to 6-week-old female athymic nude mice. Two weeks after initial implantation, the animals were placed into two experimental groups. Mice were treated with intraperitoneal injections of (a) control (saline and Intralipidor (b) honokiol, at 3 mg/mouse/day in 20% Intralipid (Baxter Healthcare, Deerfield, IL, USA), 3 times per week for the duration of the experiment. Tumors were measured by using vernier calipers, with tumor volume calculated by using the formula (V = a/2 × b2), where V is the tumor volume in cubic millimeters, and *a *and *b *are the largest and smallest diameters in millimeters, respectively. All animals were killed after 4 weeks of treatment. Tumors were collected; weighed, fixed in 10% neutral-buffered formalin; and subjected to further analysis with immunohistochemistry.

### Immunohistochemical analysis

We used tumor sections to determine the effect of honokiol on expression of p-AMPK, LKB1, and Ki-67 by immunohistochemistry. Immunohistochemistry was performed essentially as described by us previously for other proteins [25,26]. At least four nonoverlapping representative images from each tumor section from five mice of each group were captured by using ImagePro software for quantitation of p-AMPK, LKB1, and Ki-67 expression. Total cell lysates were prepared from tumor samples and subjected to immunoblot analysis. All animal studies were conducted in accordance with the guidelines of University ACUC.

### Statistical analysis

All experiments were performed thrice in triplicates. Statistical analysis was performed by using Microsoft Excel software. Significant differences were analyzed by using the Student *t *test and two-tailed distribution. Data were considered to be statistically significant if *P *< 0.05. Data were expressed as mean ± SEM between triplicate experiments performed thrice.

## Results

### Honokiol treatment inhibits clonogenicity, migration, and invasion of breast cancer cells

**Figure 1:**
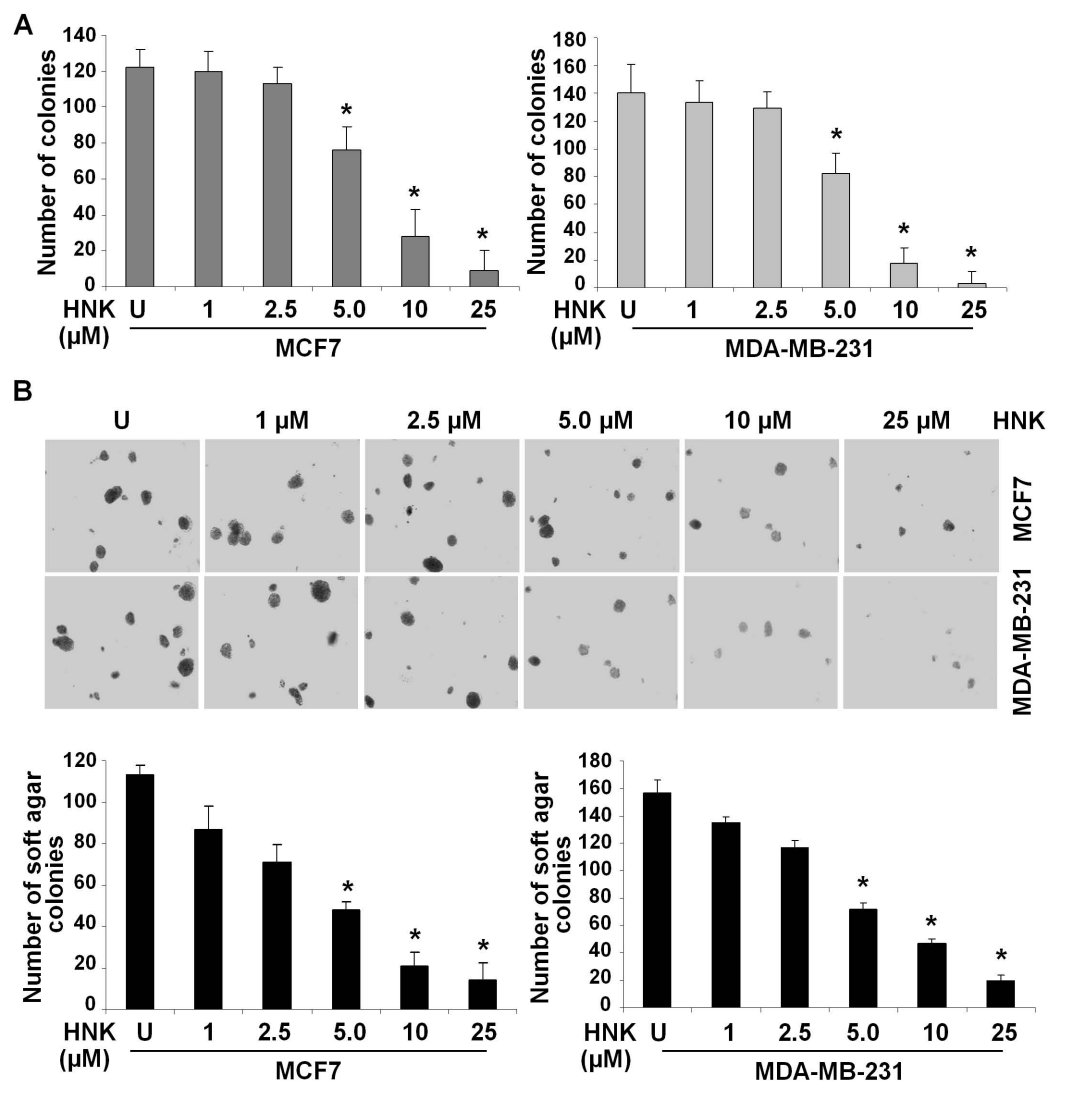
**Honokiol inhibits clonogenicity and anchorage-independent growth of breast cancer cells**. **(a) **MCF7 and MDA-MB-231 cells were treated with various concentrations of honokiol (HNK) (as indicated) and subjected to clonogenicity assay. U, untreated cells. Colonies containing > 50 normal-appearing cells were counted. *P < 0.005, compared with untreated controls. **(b) **Breast cancer cells were subjected to soft-agar colony-formation assay in the presence of various concentrations of honokiol for 3 weeks. Results are expressed as average number of colonies counted (in six microfields). *P < 0.001, compared with untreated controls.

Growth-inhibition and apoptosis-induction properties of honokiol have been reported in several cancer cell lines [3,9-12,18]. In the current study, two breast cancer cell lines, MCF7 and MDA-MB-231, were treated with various concentrations ranging from 1 μ*M *to 25 μ*M *honokiol and subjected to clonogenicity (Figure 1a) and anchorage-independent growth assay (Figure 1b). Dose-dependent and statistically significant inhibition of clonogenicity and soft-agar colony formation was observed in the presence of honokiol. Treatment with 5 μ*M *honokiol resulted in ~50% to 60% inhibition in clonogenicity and soft-agar colony formation, whereas higher concentrations (10 and 25 μ*M*) were more inhibitory (Figure 1a, b). We further examined the effect of honokiol on the growth of HCC1806 breast cancer cells, which harbor an LKB1 homozygous mutation, by using clonogenicity and soft-agar colony formation assay. Our studies show that honokiol does not inhibit the growth of HCC-1806 cells (Additional file 1). These results indicate that LKB1 might be an integral molecule for honokiol-mediated growth inhibition.

**Figure 2:**
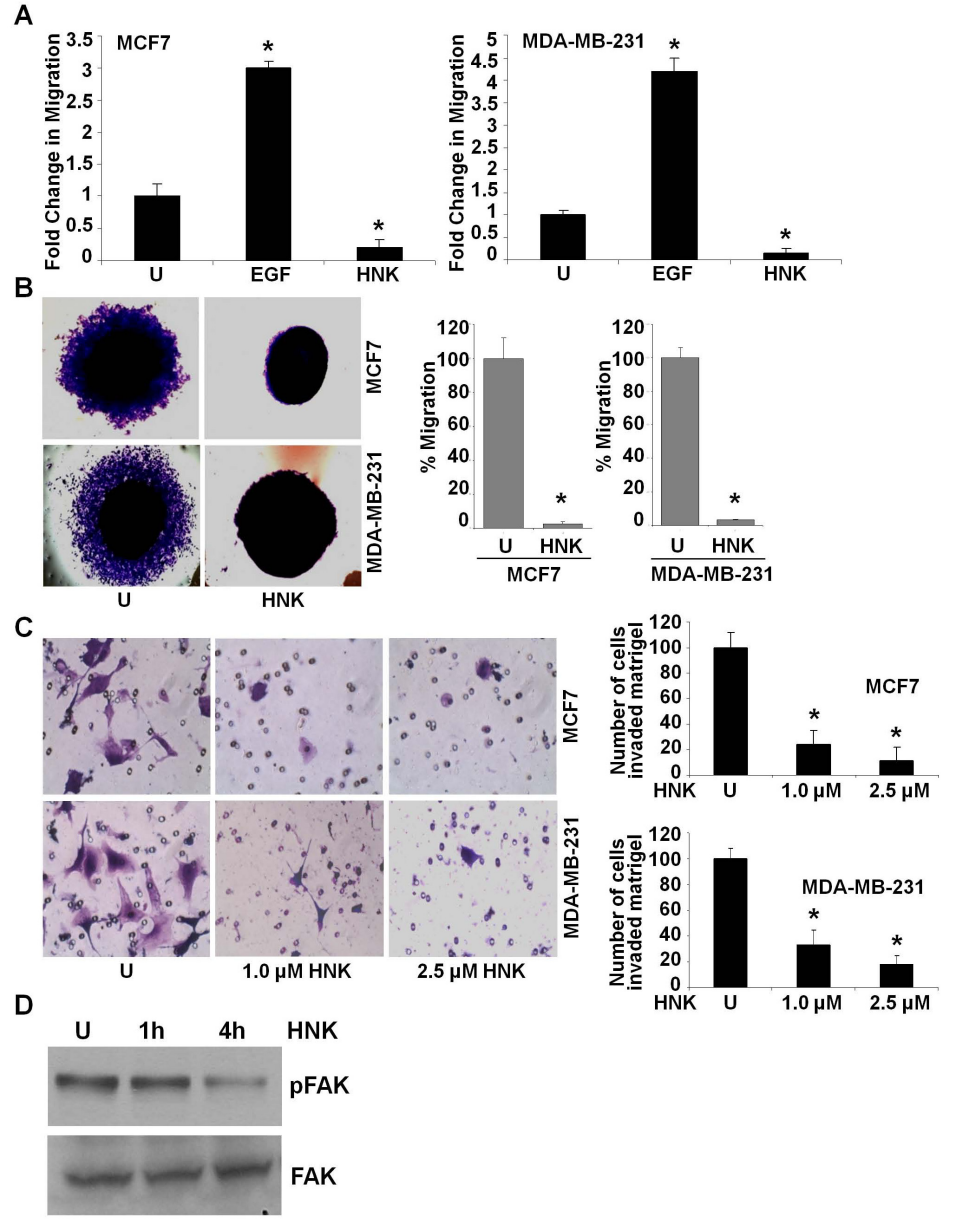
**Honokiol inhibits migration and invasion of breast cancer cells**. **(a) **MCF7 and MDA-MB-231 cells were subjected to scratch-migration assay. Culture media were replaced with media containing honokiol (2.5 μ*M*) or untreated media (U). The 100-ng/ml epidermal growth factor (EGF) treatment was used as positive control. The plates were photographed at the identical location of the initial image (0 hours) at 24 hours. The results shown are representative of three independent experiments performed in triplicate. The histogram shows the fold change in migration. *P < 0.01, compared with untreated controls. **(b) **MCF7 and MDA-MB-231 cells were subjected to spheroid-migration assay. Culture media were replaced with media containing honokiol (2.5 μ*M*) or untreated media (U). The spheroids were photographed 48 hours after treatment. The results shown are representative of three independent experiments performed in triplicate. The histograms show percentage migration. *P < 0.01, compared with untreated controls. **(c) **MCF7 and MDA-MB-231 cells were cultured in Matrigel invasion chambers followed by treatment with honokiol (HNK, 1.0, 2.5 μ*M*) for 24 hours, as indicated. U, untreated controls. The number of cells that invaded through the Matrigel was counted in five different regions. The slides were blinded to remove counting bias. The histograms show the mean of three independent experiments performed in triplicate. *P < 0.005, compared with untreated controls. **(d) **Breast cancer cells (MDA-MB-231) were treated with honokiol (HNK, 2.5 μ*M*) for indicated time intervals. U, untreated cells. Total protein was isolated, and equal amounts of proteins were resolved with SDS-PAGE and subjected to immunoblot analysis by using specific antibodies for phosphorylated FAK. The membranes were reblotted by using total FAK antibodies as controls. The blots are representative of multiple independent experiments.

Cancer progression is a multistep process that involves invasion of basement membrane by tumor cells and migration to points far from a given primary tumor mass, leading to metastasis [29]. We examined the effect of honokiol on breast cancer cell migration and invasion by using scratch migration, electric-cell-substrate impedance sensing (ECIS)-based migration, spheroid migration, and Matrigel invasion assays. Honokiol treatment resulted in inhibition of migration of breast cancer cells (Figure 2a) in comparison with untreated cells. For quantitative determination of alteration in the migration potential of breast cancer cells on treatment with honokiol, we performed a quantitative real-time impedance assay by using an ECIS-based technique. As expected, confluent cells showed high resistance values. Confluent cells were subjected to a high-voltage pulse that resulted in decrease in resistance, indicating death and detachment of cells present on the small active electrode. Cells were left untreated or treated with honokiol, and changes in resistance were recorded for 24 hours. Control untreated cells showed an increase in resistance, showing increased migration of cells surrounding the small active electrode that were not submitted to the elevated voltage pulse to reach the resistance values of the nonwounded cells at the start of the experiment. Honokiol-treated cells showed a decrease in resistance, indicating decreased migration. Notably, honokiol-treated cells never reached the values of nonwounded cells, showing significant inhibition of migration potential (Additional file 2). We examined the effect of honokiol treatment on the migratory capacity of MCF7 and MDA-MB-231 cells spheroids. Significant migration of MCF7 and MDA-MB-231 cells from the spheroids was seen under untreated conditions. Honokiol treatment resulted in inhibition of migration of cells from spheroids (Figure 2b). Next, we performed Matrigel invasion assay to examine the effect of honokiol on the invasion potential of breast carcinoma cells. As evident from Figure 2c, honokiol treatment decreased invasion of breast cancer cells through Matrigel in comparison with untreated cells. Activation of FAK has been shown to regulate cancer cell migration and invasion through distinct pathways by promoting the dynamic regulation of focal adhesion and peripheral actin structures [30-32] and matrix metalloproteinases (MMPs)-mediated matrix degradation [33]. We examined whether honokiol treatment affects FAK activation to inhibit migration and invasion of breast cancer cells. Honokiol treatment inhibited FAK phosphorylation in breast cancer cells, indicating the involvement of FAK activation in honokiol-mediated inhibition of migration and invasion potential of breast cancer cells (Figure 2d). Collectively, these results show that honokiol treatment can effectively inhibit clonogenicity, anchorage-independent colony formation, migration, and invasion of breast carcinoma cells.

### Honokiol-induced AMPK activation plays an integral role in honokiol-mediated inhibition of mTOR activity and migration potential of cells

**Figure 3:**
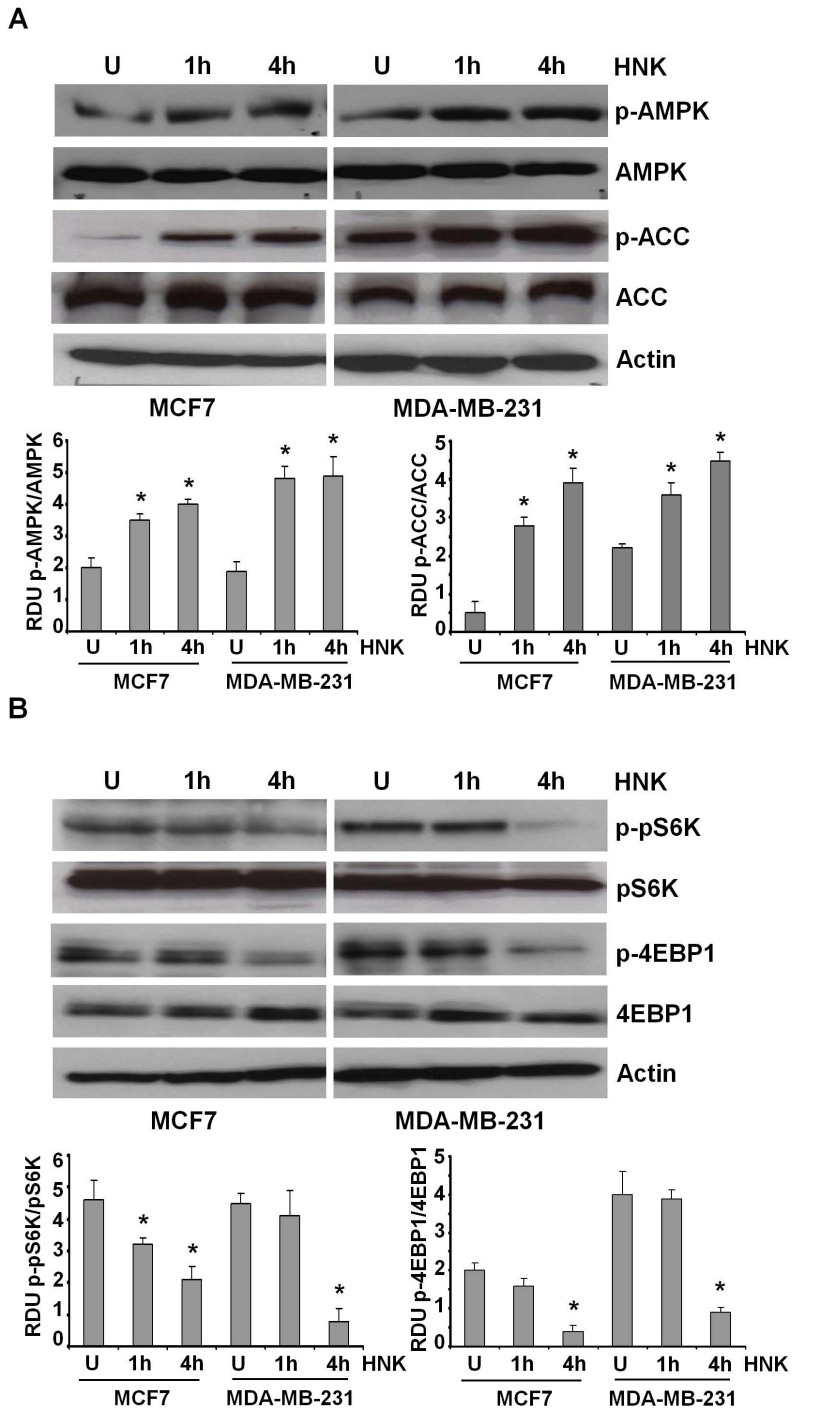
**Honokiol activates AMPK and inhibits pS6K and 4EBP1 phosphorylation in breast cancer cells**. **(a) **MCF7 and MDA-MB-231 cells were treated with honokiol (HNK, 2.5 μ*M*) for indicated time intervals. U, untreated cells. Total protein was isolated, and equal amounts of proteins were resolved with SDS-PAGE and subjected to immunoblot analysis by using specific antibodies for phosphorylated AMPK (pAMPK-Thr 172) and phosphorylated ACC (pACC). The membranes were reblotted by using total AMPK and ACC antibodies as controls. The blots are representative of multiple independent experiments. The histogram is the mean of densitometric analysis showing relative density units (RDUs) of the Western blot signal for pAMPK and pACC normalized to total AMPK or ACC in three separate experiments. *P < 0.005, compared with untreated controls. **(b) **Breast cancer cells were treated with honokiol as in **(a) **and subjected to immunoblot analysis by using specific antibodies for phosphorylated pS6K (p-pS6K) and phosphorylated 4EBP1 (p-4EBP1). The membranes were reblotted by using total pS6K and p-4EBP1 antibodies as controls. The blots are representative of multiple independent experiments. The histogram is the mean of densitometric analysis showing relative density units (RDUs) of the Western blot signal for p-pS6K and p-4EBP1 normalized to total pS6K or 4EBP1 in three separate experiments. *P < 0.001, compared with untreated controls.

Honokiol modulates multiple pathways (nuclear factor (NF)-κB, ERK, Akt, and JNK) in a cellular process and target-tissue-dependent manner [7,9-11,19]. AMP-activated protein kinase (AMPK) is a serine/threonine protein kinase that acts as a master sensor of cellular energy balance in mammalian cells by regulating glucose and lipid metabolism [34]. Recent studies have implicated AMPK as an important factor in cancer cell growth and migration [35,36]. Thus, we sought to determine the effect of honokiol on AMPK phosphorylation and activation. Honokiol treatment stimulated phosphorylation of AMPK at Thr-172 in MCF7 and MDA-MB-231 cells. Honokiol had no effect on total AMPK protein expression levels (Figure 3a). AMPK phosphorylation at Thr-172 has been widely associated with its activation [37]. Once activated, AMPK directly phosphorylates and inactivates a number of ATP-consuming metabolic enzymes including acetyl-coenzyme A carboxylase (ACC) [38]. We examined the phosphorylation of ACC to evaluate AMPK activity with honokiol treatment. Increased phosphorylation of ACC in MCF7 and MDA-MB-231 cells was observed in response to honokiol treatment as compared with untreated cells, whereas total ACC protein levels remain unchanged (Figure 3a). Activation of AMPK leads to suppression of mammalian target of rapamycin (mTOR) signaling, and the molecular mechanisms involve phosphorylation of tuberous sclerosis complex protein TSC2 at Thr-1227 and Ser-1345 that increases the activity of the TSC1-TSC2 complex to inhibit mTOR [37,39]. Two very well characterized and widely studied downstream effectors of mTOR are the p70 kDa ribosomal protein S6 kinase 1 (p70S6K1 or pS6K) and the eukaryotic translation initiation factor 4E (elF4E)-binding protein (4EBP1) [40]. Phosphorylation of pS6K and 4EBP1 has been widely used to assess changes in mTOR activity in response to various growth-factor pathways. We next examined the effect of honokiol on mTOR activity in breast cancer cells. Honokiol decreased phosphorylation of pS6K and 4EBP1 in both MCF7 and MDA-MB-231 cells while not affecting the total protein levels of pS6K and 4EBP1 (Figure 3b). Recent studies have shown that pS6K regulates the actin cytoskeleton by acting as an actin filament cross-linking protein and as a Rho family GTPase-activating protein [41]. It has been shown that reorganization of the actin cytoskeleton is critical for cell migration, as motile cancer cells must assemble and disassemble the actin filaments at their leading edges [42]. Depletion or inhibition of the activity of pS6K results in inhibition of actin cytoskeleton reorganization and inhibition of migration [41]. Owing to the integral role of pS6K in cancer cell migration, it is possible that honokiol-mediated inhibition of migration is mediated through pS6K inhibition.

mTOR, a key regulator of cell growth and proliferation, exists in two structurally and functionally distinct multiprotein complexes, mTORC1 and mTORC2. mTORC1 is known to activate protein synthesis and cell growth through regulating pS6K and 4E-BP1 activity, whereas mTORC2 phosphorylates Akt on Ser-473, activating cell growth, proliferation, and survival [43,44]. We found that honokiol increases AMPK activation and inhibits mTORC1 function, as evidenced by inhibition of pS6K and 4E-BP1 phosphorylation.

We next determined whether honokiol treatment modulates mTORC2 function. mTORC2 phosphorylates Akt on Ser-473. Therefore, to determine whether mTORC2 is also inhibited by honokiol under similar conditions, breast cancer cells were treated with honokiol, and the phosphorylation of Akt was determined. Honokiol did not alter Akt phosphorylation on Ser-473 in breast cancer cells (Additional file 3). These results provide evidence that honokiol only inhibits mTORC1 in breast cancer cells. Contrasting findings have been reported previously, showing reduction in Akt phosphorylation in response to honokiol treatment. Of note, MDA-MB-231 cells were treated with much higher concentrations of honokiol (60, 80, and 100 μ*M*) in this study [45]. Hence, the observed decrease in Akt phosphorylation may be due to the treatment with higher concentrations of honokiol. Honokiol inhibits breast cancer growth in a concentration-dependent manner, with higher concentrations much more inhibitory than lower concentrations (Figure 1).

**Figure 4:**
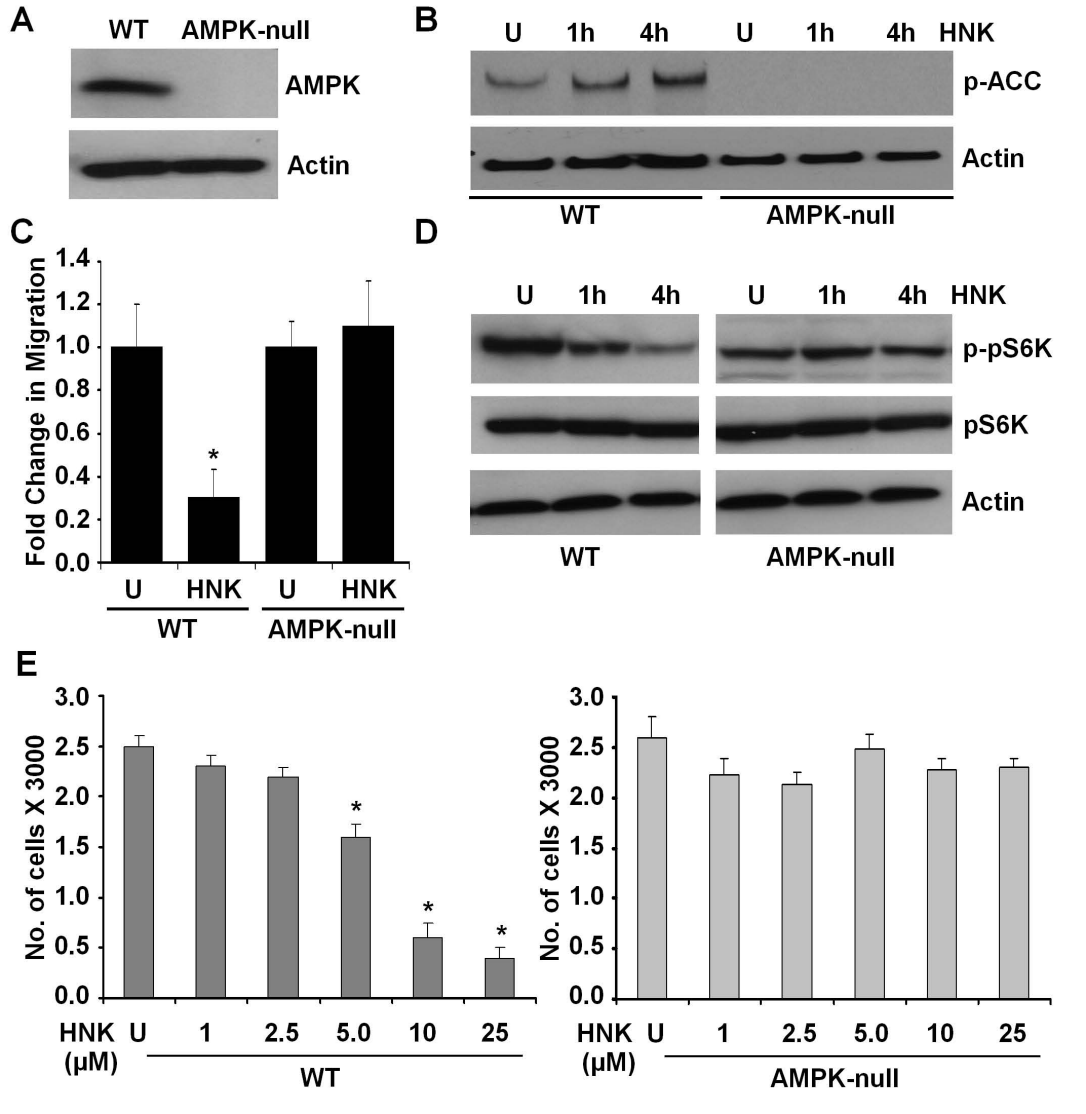
**AMPK knockdown abrogates honokiol-mediated increased phosphorylation of ACC, inhibition of phosphorylation of S6K, and inhibition of migration**. **(a) **Immunoblotting for AMPK protein by using lysates from untreated MEFs derived from AMPK-WT (WT) and AMPK-knockout mice (AMPK-null). The blot was stripped and reprobed with anti-actin antibody. **(b) **WT and AMPK-null MEFs were treated with honokiol (HNK, 2.5 μ*M*) for indicated time intervals. U, untreated cell. Total protein was isolated, and equal amounts of proteins were resolved with SDS-PAGE and subjected to immunoblot analysis by using specific antibodies for phosphorylated ACC (p-ACC). Anti-actin antibody was used as control. **(c) **WT and AMPK-null MEFs were subjected to scratch-migration assay in the presence (HNK, 2.5 μ*M*) or absence (U) of honokiol. The plates were photographed at the identical location of the initial image (0 hours) at 24 hours. The histogram shows the fold change in migration. *P < 0.001, compared with untreated controls. All the experiments were performed thrice in triplicate. **(d) **WT and AMPK-null MEFs were treated with honokiol (HNK, 2.5 μ*M*) for indicated time intervals. U, untreated cell. Total protein was isolated, and equal amounts of proteins were resolved with SDS-PAGE and subjected to immunoblot analysis by using specific antibodies for phosphorylated pS6K (p-pS6K). The membranes were reblotted by using total pS6K and actin antibody as control. **(e) **WT and AMPK-null MEFs were subjected to XTT assay in the presence (HNK) or absence (U) of honokiol, as indicated. The results shown are representative of three independent experiments performed in triplicate. *P < 0.001, compared with untreated controls.

Although our findings clearly showed the involvement of AMPK activation in the honokiol signaling network, we raised the question whether honokiol-induced inhibition of mTOR and cell migration requires AMPK protein. We used MEFs derived from AMPK-WT (WT) and AMPK knockout (AMPK-null) mice to test the potential requirement of this protein in honokiol-mediated inhibition of migration. Immunoblotting confirmed the absence of the AMPK protein in AMPK-null MEFs (Figure 4a). In agreement with the absence of AMPK protein, the AMPK-null MEFs did not show any phosphorylation of ACC, even in the presence of honokiol. AMPK-WT MEFs, conversely, exhibited honokiol-stimulated phosphorylation of ACC, indicating activation of AMPK (Figure 4b). Exposure of MEFs derived from AMPK-WT mice to honokiol resulted in inhibition of phosphorylation of pS6K, whereas the MEFs derived from the AMPK-null mice were significantly resistant to the honokiol-mediated inhibition of pS6K phosphorylation (Figure 4d).

We next asked whether AMPK is directly involved in honokiol-mediated inhibition of migration. AMPK-WT MEFs exhibited inhibition of migration in response to honokiol treatment in scratch migration as well as ECIS-based migration assay. Interestingly, honokiol treatment could not inhibit migration of AMPK-null MEFs (Figure 4c; Additional file 4). AMPK knockdown also inhibited the antiproliferative effect of honokiol (Figure 4e). These results showed that AMPK is an integral molecule in mediating the negative effects of honokiol on the mTOR axis and migration potential of cells.

### Inhibition of LKB1 abrogates honokiol-mediated modulation of AMPK and inhibition of migration and invasion of breast cancer cells

**Figure 5:**
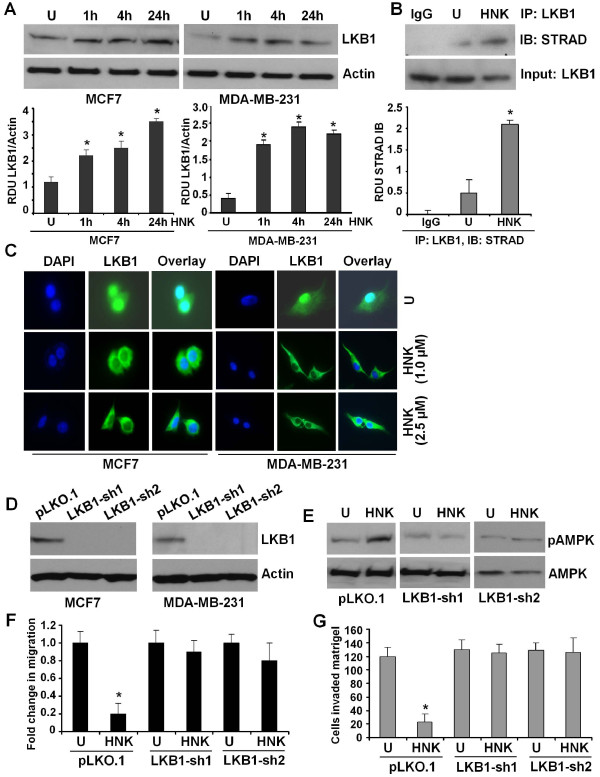
**Honokiol increases LKB1 expression, LKB1:STRAD interaction, cytosolic translocation, and depletion of LKB1 abrogates honokiol-mediated modulation of AMPK, inhibition of migration, and invasion of breast cancer cells**. **(a) **MCF7 and MDA-MB-231 cells were treated with 2.5 μ*M *honokiol for indicated time intervals. U, untreated cell. Total protein was isolated, and equal amounts of proteins were resolved with SDS-PAGE and subjected to immunoblot analysis by using specific antibodies for LKB1. The membranes were reblotted by using actin antibody as control. The blots are representative of multiple independent experiments. The histogram is the mean of densitometric analysis showing relative density units (RDUs) of the Western blot signals for LKB1 normalized to actin in three independent experiments. *P < 0.005, compared with untreated controls. **(b) **MCF7 cells were treated with 2.5 μ*M *honokiol or untreated and subjected to immunoprecipitation assay by using IgG or LKB1 antibodies, as indicated. Immunoprecipitates were analyzed by using anti-STRAD antibodies. The histogram is the mean of densitometric analysis showing relative density units (RDUs) of the Western blot signals for STRAD in three independent experiments. *P < 0.005, compared with untreated controls. **(c) **MCF7 and MDA-MB-231 cells were treated with honokiol (HNK), and LKB1 protein was analyzed with immunofluorescence by using LKB1 antibody; 4'6-diamidino-2-phenylindole staining was used to determine the nuclear localization. These results are representative of multiple independent experiments. **(d) **LKB1 was depleted in MCF7 and MDA-MB-231 cells by using two different lentiviral LKB1 short-hairpin RNA (shRNA1 and shRNA2) constructs and a negative control construct that was created in the same vector system (pLKO.1). Stable pools of LKB1-depleted (LKB1^shRNA^) and vector control (pLKO.1) cells were used for total protein isolation, and equal amounts of proteins were subjected to immunoblot analysis by using specific antibodies for LKB1. Actin was used as control. **(e) **MDA-MB-231-LKB1^shRNA ^(LKB1-sh1 and LKB1-sh2) and MDA-MB-231-pLKO.1 (pLKO.1) cells were treated with honokiol (HNK, 2.5 μ*M*), and phosphorylation of AMPK was analyzed with Western blot analysis. Total AMPK antibody was used as control. **(f) **MDA-MB-231-LKB1^shRNA ^(LKB1-sh1 and LKB1-sh2) and MDA-MB-231-pLKO.1 (pLKO.1) cells were grown to confluence, scratched with a pipette tip, and photographed immediately after scratching (0 hours). Culture media were replaced with media containing honokiol (HNK, 2.5 μ*M*) or untreated media (U). The plates were photographed at the identical location of the initial image (0 hours) at 24 hours. The results shown are representative of three independent experiments performed in triplicate. **(g) **MDA-MB-231-LKB1^shRNA ^(LKB1-sh1 and LKB1-sh2) and MDA-MB-231-pLKO.1 (pLKO.1) cells were cultured in Matrigel invasion chambers followed by treatment with honokiol (HNK, 2.5 μ*M*) for 24 hours. The number of cells that invaded through the Matrigel was counted in five different regions. The slides were blinded to remove counting bias. The result shows the mean of three independent experiments performed in triplicate. *P < 0.005, compared with untreated controls.

The tumor suppressor LKB1 (also known as Stk11) is an evolutionarily conserved serine/threonine protein kinase that has a broad range of cellular functions, including tumor suppression, cell polarity, cell-cycle regulation, and promotion of apoptosis [46,47]. LKB1 has recently been identified as a critical upstream kinase for AMPK, regulating its activity. Intriguingly, we found that honokiol increases expression of tumor suppressor LKB1 in MCF7 and MDA-MB-231 cells, with a significant increase at 1 hour of treatment with maximal expression at 24 hours in MCF7 cells and at 4 hours in MDA-MB-231 cells (Figure 5a). Variable expression of LKB1 in MDA-MB-231 breast cancer cells has been reported [48,49]. We recently procured MDA-MB-231 cells from various established breast cancer research laboratories and analyzed the expression and functional status of LKB1. Our data unequivocally showed the presence of functional LKB1 in MDA-MB-231 cells [22]. Human LKB1 is both nuclear and cytoplasmic, but a mutant of LKB1 lacking the nuclear localization signal still retains the ability to suppress cell growth, suggesting that the cytosolic pool of LKB1 plays an important role in mediating its tumor-suppressor properties [50,51]. STRAD (Ste20-related adaptor) protein has been shown to form a complex in which STRAD activates LKB1, resulting in cytoplasmic translocation of LKB1 [47]. We investigated the effect of honokiol on the formation of the LKB1-STRAD complex in breast cancer cells. To address this question, breast cancer cells were treated with honokiol followed by immunoprecipitation with LKB1 antibodies. Immunoprecipitated protein complexes were analyzed for the presence of STRAD by using Western blot analysis. Higher levels of STRAD immunoprecipitated with LKB1 in the presence of honokiol indicated increased formation of the LKB1-STRAD complex (Figure 5b). Immunostaining of honokiol-treated MCF7 and MDA-MB-231 cells revealed that honokiol treatment increases cytoplasmic accumulation of LKB1. LKB1 was localized predominantly in the nucleus in untreated breast cancer cells, although cytoplasmic LKB1 expression was also detected (Figure 5c). Control experiments with secondary antibody (results not shown) gave an extremely faint background staining that was distributed uniformly throughout the cells, irrespective of the treatment. Studies on the subcellular localization of LKB1 have indicated a wide variety of localization patterns. Mouse LKB1 was found to be predominantly nuclear, whereas *Caenorhabditis elegans *PAR-4 and *Xenopus *XEEK1 were detected exclusively in the cytoplasm [52-54]. Human LKB1 has been detected to be both nuclear and cytoplasmic in several cell types [55,56]. Although LKB1 expression is exclusively cytoplasmic in lung and pancreatic cancer [57,58], gastrointestinal hamartomatous polyps from Peutz-Jeghers syndrome patients, head and neck squamous cell carcinoma, invasive lobular breast carcinoma, and solid papillary ductal carcinoma *in situ *breast cancer show both cytoplasmic and nuclear LKB1 expression [59-61]. Studies in adult rat primary cardiomyocytes and C2C12 myoblasts showed that LKB1 was located predominantly in nucleus and undergoes cytoplasmic localization in various stimulations [62-64]. *In vitro *studies suggest that nuclear LKB1 regulates cell-cycle progression and acts as a transcription factor [65,66], whereas cytoplasmic LKB1 participates in controlling energy metabolism and cell polarity [67]. It is not completely understood how subcellular localization of LKB1 affects its tumor-suppressor function and activation of other signaling pathways *in vivo*.

We raised the question whether LKB1 plays an important regulatory role in honokiol- mediated modulation of AMPK and inhibition of migration and invasion of breast cancer cells. To address these questions, we used LKB1^shRNA ^lentivirus and puromycin to select for stable pools of MCF7 and MDA-MB-231 cells with LKB1 depletion. We analyzed pLKO.1 and LKB1^shRNA ^stable MCF7 and MDA-MB-231 cell pools for LKB1 protein expression with immunoblot analysis and found that LKB1 protein expression was significantly reduced in LKB1^shRNA ^cells (shRNA1 and shRNA2) as compared with pLKO.1 control cells (Figure 5d). pLKO.1 and LKB1^shRNA ^cells were treated with honokiol, and phosphorylation of AMPK was determined by using Western blot analysis. We found that honokiol increased phosphorylation of AMPK in pLKO.1 cells. Intriguingly, displaying a crucial role of LKB1, honokiol treatment did not change the phosphorylation levels of AMPK in LKB1^shRNA ^cells (Figure 5e). Invasion and migration are the key biologic features of malignant behavior of carcinoma cells [29]. In addition to examining the effect of LKB1 depletion on honokiol-induced modulation of AMPK, we also examined the requirement of LKB1 in honokiol-mediated inhibition of metastatic properties of breast cancer cells. As evident from Figure 5f, honokiol treatment efficiently inhibited migration of pLKO.1 cells, whereas untreated pLKO.1 cells showed increased migration. Our results showed that LKB1^shRNA ^cells exhibited increased migration in the absence of honokiol treatment. Interestingly, honokiol treatment did not inhibit the migration of LKB1^shRNA ^cells (Figure 5f). We next examined the effect of honokiol on invasion potential of pLKO.1 and LKB1^shRNA ^cells and found that honokiol inhibited invasion of pLKO.1 cells, whereas LKB1^shRNA ^cells were not affected by honokiol treatment (Figure 5g). These results collectively show that honokiol-induced LKB1 overexpression is indeed a crucial component of the signaling machinery used by honokiol in modulating the AMPK-S6K axis and inhibiting the metastatic properties of breast cancer cells.

### Honokiol treatment inhibits breast-tumor progression in athymic nude mice

**Figure 6:**
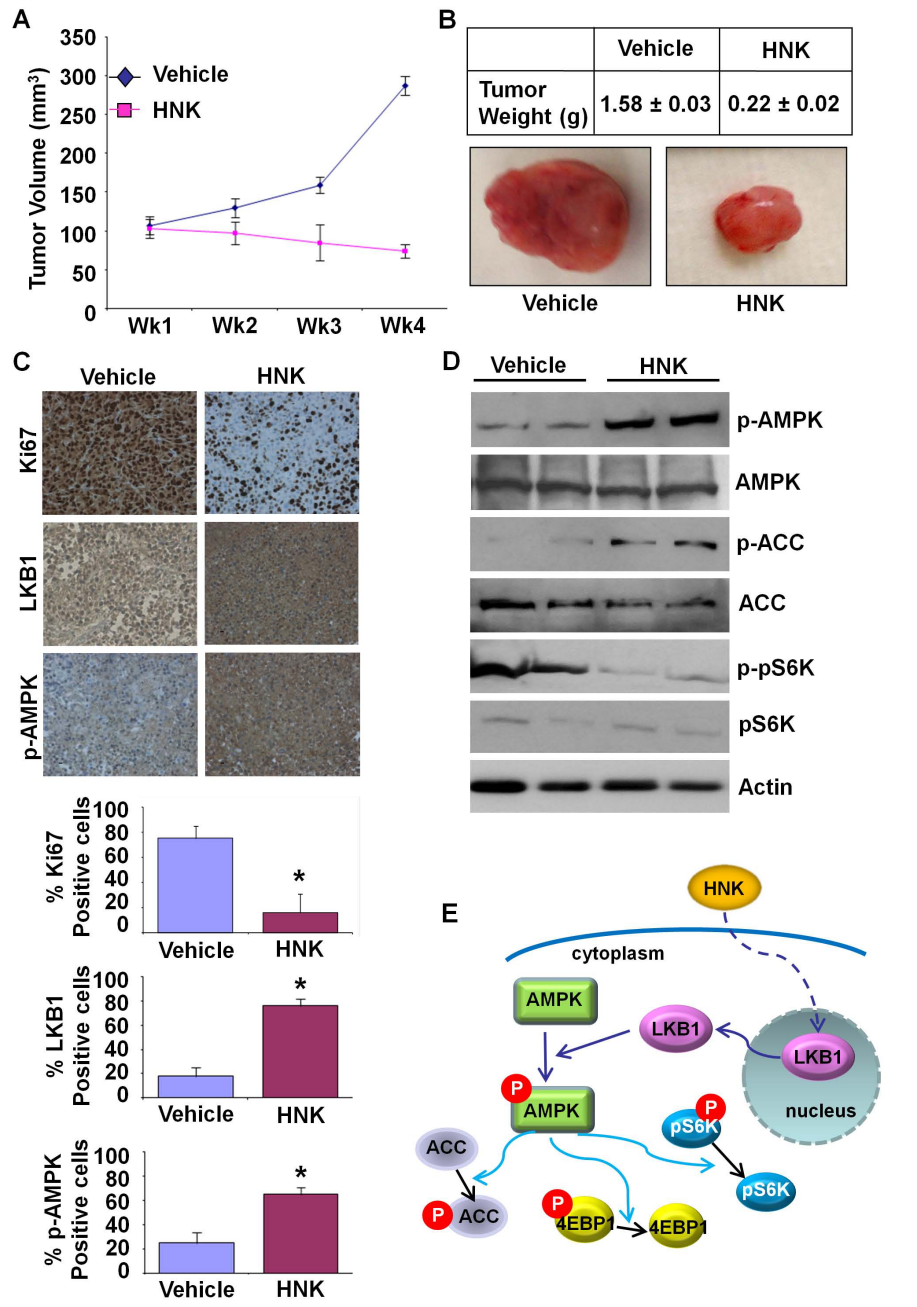
**Honokiol treatment inhibited breast tumor growth in nude mice**. MDA-MB-231 cells-derived tumors were developed in nude mice and treated with vehicle or honokiol (HNK). **(a) **Tumor growth was monitored by measuring the tumor volume for 4 weeks (eight mice per group). **(b) **At the end of 6 weeks, tumors were collected, measured, weighed, and photographed. Honokiol treatment inhibited tumor size as compare with vehicle treatment. Average tumor weight and representative tumor images are shown here. **(c) **Tumor samples were subjected to immunohistochemical analysis by using LKB1, p-AMPK, and Ki67 antibodies. Honokiol (HNK) treatment decreased the expression of Ki-67, increased expression of LKB1 and pAMPK, as compared with vehicle treatment. Bar diagrams show quantitation of protein expression in tumors from vehicle- and honokiol-treated mice. Columns, mean (*n *= 8); bar, SD. *Significantly different (*P *< 0.005) compared with control. **(d) **Tumor lysates (from two different tumors from each set) were subjected to immunoblot analysis by using phospho-AMPK (p-AMPK), AMPK, phospho-ACC (p-ACC), ACC, phospho-pS6K, pS6K antibodies. Actin antibody was used as control. **(e) **A model of honokiol (HNK)-stimulated AMPK activation in breast cancer cells. Honokiol stimulation induces LKB1 translocation from the nucleus into cytosol and phosphorylates AMPK, leading to increased phosphorylation of ACC and decreased phosphorylation of pS6K and 4EBP1.

We investigated the physiological relevance of our *in vitro *findings by evaluating whether honokiol has any suppressive effects on the development of breast carcinoma in nude mouse models and the involvement of the LKB1-AMPK axis. In the experimental group treated with honokiol, the rate of tumor growth was significantly inhibited, and the tumor size and weight were significantly reduced compared with control group (Figure 6a, b). The immunohistochemical assessment of tumor proliferation showed higher Ki-67 in the control group as compared with the honokiol-treated group (Figure 6c). In our *in vitro *analyses, we discovered the involvement and requirement of the LKB1-AMPK axis in biologic functions of honokiol. We examined the expression of LKB1 and p-AMPK in tumors treated with honokiol. Tumors treated with honokiol displayed higher levels of phosphorylated AMPK and LKB1 (Figure 6c). In addition, we examined the expression levels of phosphorylated and unphosphorylated AMPK, ACC as well as S6K, in honokiol-treated and vehicle-treated mice. We found higher levels of phosphorylated AMPK and ACC in honokiol-treated tumors as compared with vehicle-treated controls. Honokiol-treated tumors showed lower levels of phosphorylated S6K, whereas vehicle-treated controls exhibited high levels of phosphorylated S6K (Figure 6d). These data presented direct *in vivo *evidence of the involvement of LKB1-AMPK activation and the subsequent inhibition of pS6K in honokiol function (Figure 6e).

## Discussion

The antitumor activity of honokiol, a natural product derived from magnolia plant and used in traditional Asian medicine, has been reported in various preclinical models [3]. In the current study, we investigated the potential of honokiol in the inhibition of migration and invasion of breast cancer cells and the underlying molecular mechanisms. The following novel findings are reported in this study: (i) honokiol treatment inhibits malignant properties such as invasion and migration of breast cancer cells; (ii) honokiol stimulates AMPK phosphorylation and activity while reducing mTOR activity, as evidenced by reduced phosphorylation of pS6K and 4EBP1; (iii) AMPK protein is required for honokiol-mediated inhibition of pS6K and 4EBP1; (iv) honokiol increases the expression and cytosolic localization of tumor suppressor LKB1, which is an essential effector molecule to mediate the honokiol effect on the AMPK-pS6K axis and inhibition of invasion and migration of breast cancer cells; and (v) honokiol inhibits breast tumor growth and modulates the LKB1-AMPK-pS6K axis *in vivo*. Our results show that honokiol treatment significantly inhibits malignant properties of breast cancer cells through modulation of the LKB1-AMPK-pS6K axis; thus using honokiol may be a suitable therapeutic strategy for metastatic breast cancer.

Many bioactive molecules and their synthetic analogues have been reported to demonstrate activity against breast cancer [68-71]. Although the lower toxicity associated with bioactive molecules is a much desired quality, their limited bioavailability hinders further development. Honokiol exhibits a desirable spectrum of bioavailability, in contrast with many other natural products [3]. The development of other polyphenolic agents has been obstructed by poor absorption and rapid excretion [72]. Honokiol does not have this disability, as significant systemic levels of honokiol can be obtained in preclinical models, and it can cross the blood-brain barrier [73]. These qualities of honokiol make it a promising small-molecular-weight natural anticancer agent. Indeed, honokiol has been found to alter many molecular targets in various cancer models to inhibit tumor cell growth and survival [3,6,9,10,12,19]. One of the major findings of this study is that the LKB1-AMPK pathway plays a major role in mediating the effect of honokiol effect on migration and invasion of breast cancer cells.

AMPK, a master sensor of cellular energy balance in mammalian cells, regulates glucose and lipid metabolism [34]. Biochemical regulation of serine/threonine protein kinase AMPK activation occurs through multiple mechanisms [37]. AMPK undergoes a conformational change in response to direct binding of AMP to its nucleotide-binding domain, exposing the activation loop of the catalytic kinase subunit. LKB1 phosphorylates a critical threonine in this activation loop to activate AMPK. Dephosphorylation by protein phosphatases also plays an important role in regulating AMPK activity [47]. Genetic depletion of LKB1 in mouse embryonic fibroblasts (MEFs) results in a loss of AMPK activation after energy stresses that increase AMP [37], showing the requirement of LKB1 in AMPK activation. We found that honokiol increases AMPK activation, which can be efficiently inhibited by the silencing of LKB1. AMPK represents a pivotal point in the mTOR pathway regulating a vast range of cellular activities, including transcription, translation, cell size, mRNA turnover, protein stability, ribosomal biogenesis, and cytoskeletal organization [37]. Besides being directly activated by tumor-suppressor LKB1, AMPK itself regulates the activation of two other tumor suppressors, TSC1 and TSC2, which are critical regulators of Rheb and mTOR [37]. We found that AMPK knockdown inhibits honokiol-mediated mTOR inhibition. Honokiol-mediated inhibition of mTOR also suggests that honokiol and its derivatives may prove excellent candidates as targeted therapies for carcinomas characterized by hyperactive mTOR signaling.

LKB1 kinase is a tumor suppressor and a key determinant in the Peutz-Jeghers syndrome, an inherited susceptibility to gastrointestinal, lung, pancreatic, and breast cancer [47,74]. Inactivation of the *LKB1 *gene has been shown in a subset of sporadic lung and pancreatic cancer. Although the loss of LKB1 expression is not commonly observed in human breast carcinoma, it certainly correlates with high-grade DCIS and high-grade invasive ductal carcinoma [61]. It is important to note that LKB1 expression was not abrogated in pure DCIS cases but only in the DCIS associated with invasion, indicating that loss of LKB1 could potentially promote invasion. Supporting this notion, low LKB1 protein levels have been reported to correlate with poor prognosis in breast carcinoma [48]. Our studies show that honokiol treatment increases the expression and cytosolic localization of LKB1 in breast xenograft tumors and inhibits tumor growth. LKB1 is localized predominantly in the nucleus, translocating to the cytosol, either by forming a heterotrimeric complex with STRAD (ste20-related adaptor protein) and MO25 (mouse protein 25) or by associating with LIP1 (LKB1-interacting protein), to exert its biologic functions [50,52,55,75,76]. The cytoplasmic pool of LKB1 plays an important role in mediating its tumor-suppressor properties. Wild-type LKB1, when co-expressed with STRAD and MO25, exhibits increased cytoplasmic localization, whereas mutant LKB1, unable to interact with STRAD and MO25, remains in the nucleus [75,77]. Promotion of cytosolic translocation of LKB1 is a common mechanism to activate downstream LKB1 functions, as AMPK activation by metformin, peroxynitrile, or adiponectin also involves LKB1 cytosolic translocation [22,64,78-80]. Honokiol treatment increases LKB1-STRAD complex formation in addition to overexpression of LKB1, thus increasing the functional pool of LKB1. Our study shows for the first time that honokiol stimulates the cytosolic translocation of LKB1 in breast cancer cells.

## Conclusions

We uncovered a novel mechanism by which honokiol inhibits invasion and migration of breast cancer cells, which involves enhanced expression and cytosolic localization of LKB1 and AMPK activation. We also demonstrated the requirement of LKB1 and AMPK in honokiol-mediated inhibition of migration and invasion of breast cancer cells. Our results thus provide new insight into the mechanisms by which honokiol, a promising anticancer agent, inhibits breast carcinogenesis.

## Abbreviations

4EBP1: 4E binding protein 1; ACC: acetyl-coenzyme A carboxylase; AMPK: AMP-activated protein kinase; ECIS: electric cell-substrate impedance sensing; ERK: extracellular signal-regulated kinase; LKB1: liver kinase B1; MEFs: mouse embryonic fibroblasts; pS6K: p70S6kinase; WT: wild type.

## Conflicting interests

AN, MYB, NKS, and DS declare no conflict of interest. JLA is listed as an inventor on patents filed by Emory University. Emory has licensed its honokiol technologies to Naturopathic Pharmacy. JLA has received stock in Naturopathic Pharmacy, which, to the best of our knowledge, is not publically traded.

## Authors' contributions

AN performed experiments at the Sharma Lab; JLA and MYB standardized and performed honokiol isolation; NKS and DS designed research and wrote the article. All authors read and approved the final manuscript.

## Supplementary Material

Additional file 1 **Figure S1. Honokiol inhibits clonogenicity and anchorage-independent growth of HCC-1806 breast cancer cells. (a) **HCC-1806 breast cancer cells were treated with various concentrations of honokiol (HNK) (as indicated) and subjected to clonogenicity assay. U, untreated cells. Colonies containing > 50 normal-appearing cells were counted. *P < 0.005, compared with untreated controls. **(b) **HCC-1806 breast cancer cells were subjected to soft-agar colony-formation assay in the presence of various concentrations of honokiol for 3 weeks. U, untreated cells. Results are expressed as average number of colonies counted (in six microfields). *P < 0.001, compared with untreated controls. Click here for file

Additional file 2 **Figure S2. Honokiol inhibits migration and invasion of breast cancer cells**. Confluent layer of MCF7 and MDA-MB-231 breast cancer cells grown on electric cell-substrate impedance sensing (ECIS) 8W1E plates was subjected to an elevated voltage pulse of 40 kHz frequency, 3.5 V amplitude for 30 seconds to create a wound, and resistance was measured for 24 hours in the presence (HNK, 2.5 μ*M*) and absence (U) of honokiol to monitor the migration of breast cancer cells. Honokiol treatment inhibited migration of breast cancer cells in an ECIS assay. All the experiments were performed thrice in triplicate. Click here for file

Additional file 3 **Figure S3. Honokiol does not modulate Akt activation in breast cancer cells**. MCF7 and MDA-MB-231 cells were treated with honokiol (HNK, 2.5 μ*M*) for indicated time intervals. U, untreated cells. Total protein was isolated, and equal amounts of proteins were resolved with SDS-PAGE and subjected to immunoblot analysis by using specific antibodies for phosphorylated Akt. The membranes were reblotted by using total Akt antibodies as controls. The blots are representative of multiple independent experiments. Click here for file

Additional file 4 **Figure S4. AMPK knockdown abrogates honokiol-mediated inhibition of migration**. Confluent layer of WT and AMPK-null MEFs grown on electric cell-substrate impedance sensing (ECIS) 8W1E plates was subjected to an elevated voltage pulse of 40 kHz frequency, 3.5 V amplitude, for 30 seconds to create a wound, and resistance was measured for 24 hours in the presence (HNK, 2.5 μ*M*) and absence (U) of honokiol to monitor the migration of MEFs. All the experiments were performed thrice in triplicate. Click here for file
