# Erratum to: Honokiol activates AMP-activated protein kinase in breast cancer cells via LKB1-dependent pathway and inhibits breast carcinogenesis

**DOI:** 10.1186/s13058-017-0829-2

**Published:** 2017-03-28

**Authors:** Arumugam Nagalingam, Jack L. Arbiser, Michael Y. Bonner, Neeraj K. Saxena, Dipali Sharma

**Affiliations:** 1 Department of Oncology, Johns Hopkins University School of Medicine and the Sidney Kimmel Comprehensive Cancer Center at Johns Hopkins, Baltimore, MD 21231 USA; 2 Department of Dermatology, Emory University School of Medicine, Winship Cancer Institute, Atlanta, GA 30322 USA; 3 Department of Medicine, University of Maryland School of Medicine, Baltimore, MD 21201 USA

## Erratum

After the publication of this work [1], an error was noticed in Fig. 1b. An incorrect image was inadvertently shown for the soft-agar colony for MDA-MB-231 cells treated with 5.0 uM Honokiol. The correct figure is shown below. This error did not affect the findings or conclusions of the article.

**Fig. 1:**
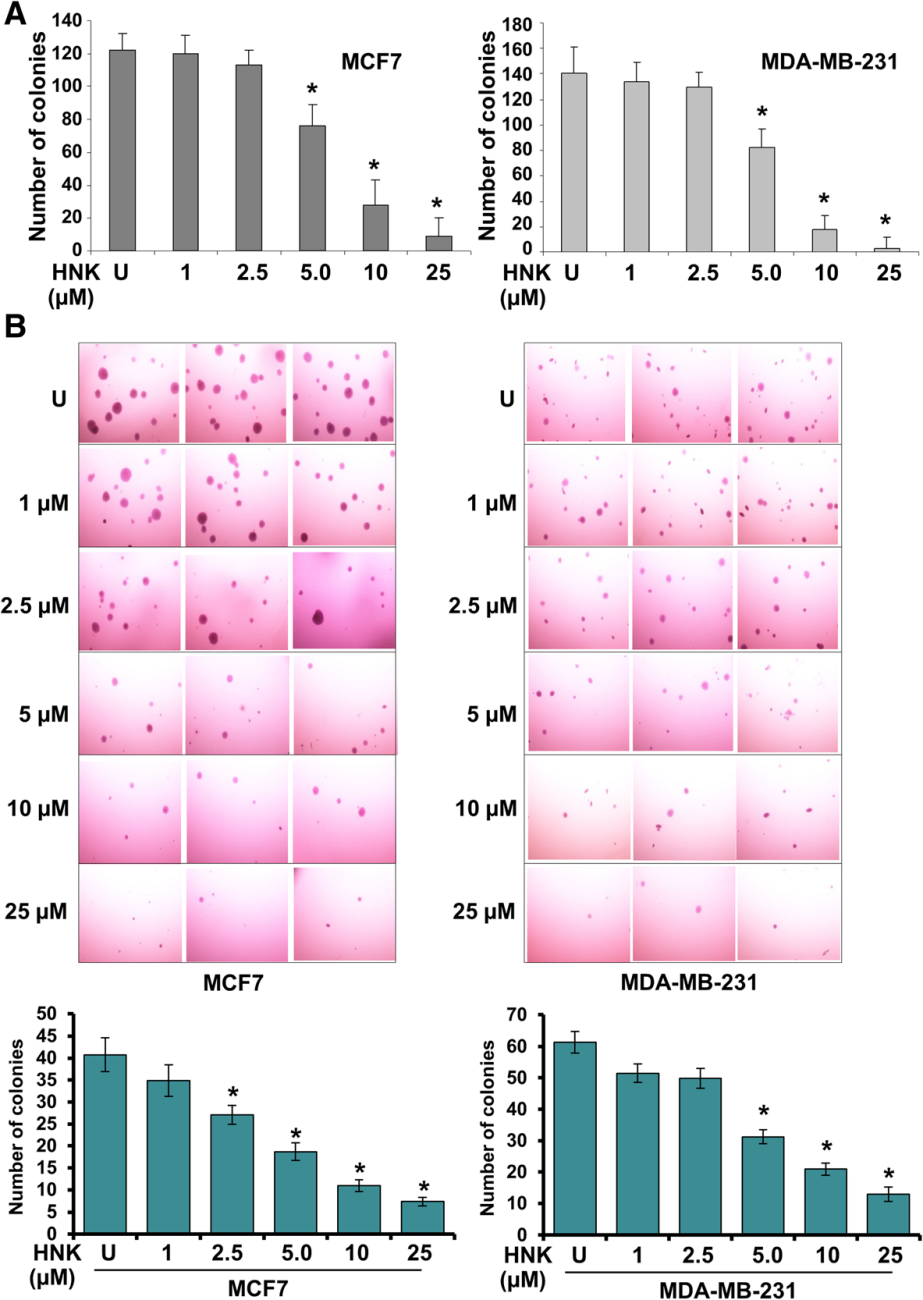
Honokiol inhibits clonogenicity and anchorage-independent growth of breast cancer cells. **a** MCF7 and MDA-MB-231 cells were treated with various concentrations of honokiol (HNK) (as indicated) and subjected to clonogenicity assay. Untreated cells, denoted with the letter “U”. Colonies containing >50 normal-appearing cells were counted. *, *P* < 0.005, compared with untreated controls. **b** Breast cancer cells were subjected to soft-agar colony-formation assay in the presence of various concentrations of honokiol for three weeks. Untreated cells are denoted with the letter “U”. Results are expressed as average number of colonies counted (in six micro-fields). *, *P* < 0.001, compared with untreated controls

## References

[1] Nagalingam A (2012). Honokiol activates AMP-activated protein kinase in breast cancer cells via an LKB1-dependent pathway and inhibits breast carcinogenesis. Breast Cancer Res.

