# Supplementary figures and images for: Honokiol activates AMP-activated protein kinase in breast cancer cells via an LKB1-dependent pathway and inhibits breast carcinogenesis
Source: Breast Cancer Res. 2012 Feb 21;14(1):R35. doi: 10.1186/bcr3128 (PMC3496153; doi:10.1186/bcr3128)

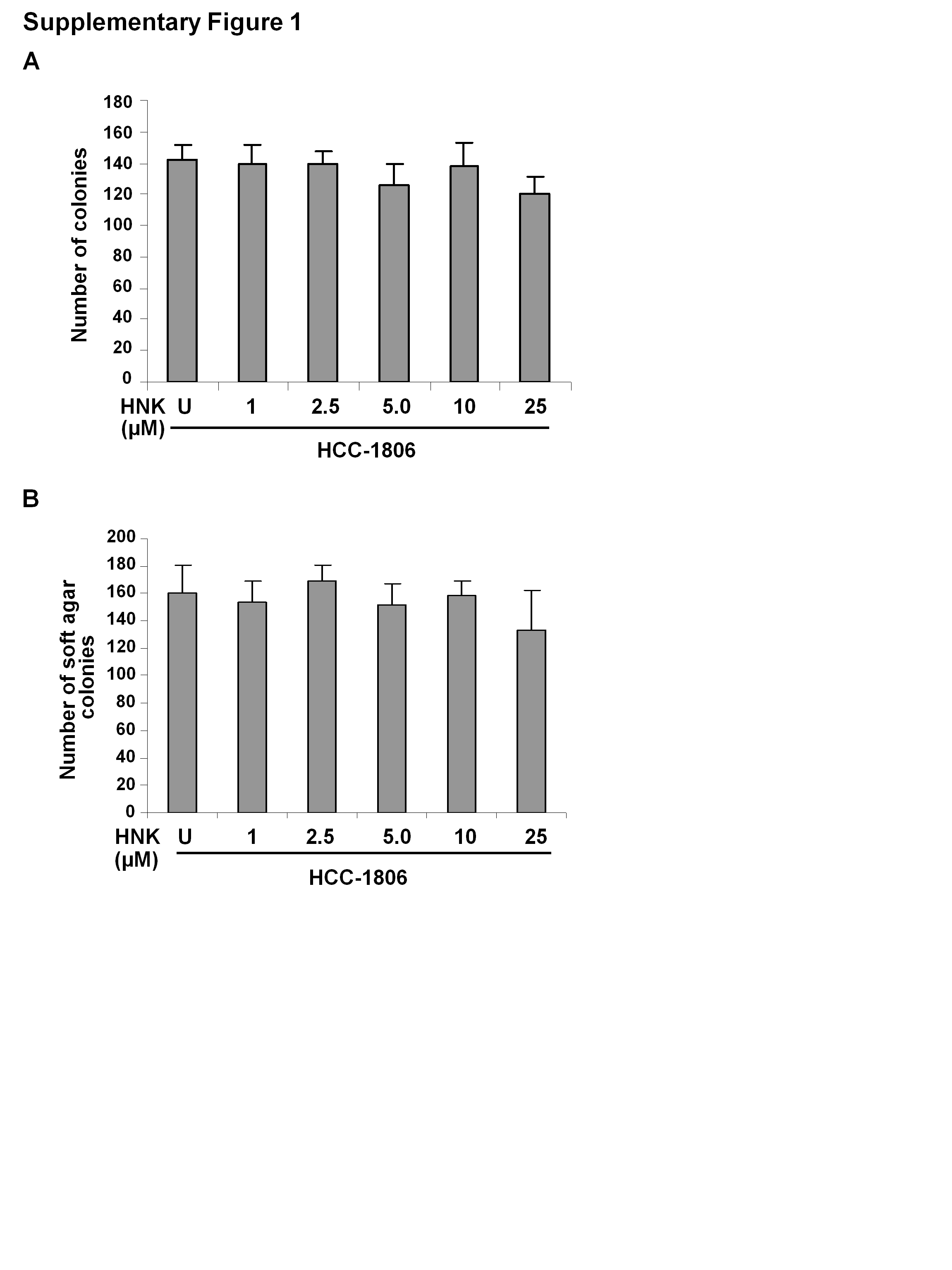

Supplement: Additional file 1 — Figure S1. Honokiol inhibits clonogenicity and anchorage-independent growth of HCC-1806 breast cancer cells. (a) HCC-1806 breast cancer cells were treated with various concentrations of honokiol (HNK) (as indicated) and subjected to clonogenicity assay. U, untreated cells. Colonies containing > 50 normal-appearing cells were counted. *P < 0.005, compared with untreated controls. (b) HCC-1806 breast cancer cells were subjected to soft-agar colony-formation assay in the presence of various concentrations of honokiol for 3 weeks. U, untreated cells. Results are expressed as average number of colonies counted (in six microfields). *P < 0.001, compared with untreated controls. [file bcr3128-S1.TIFF]

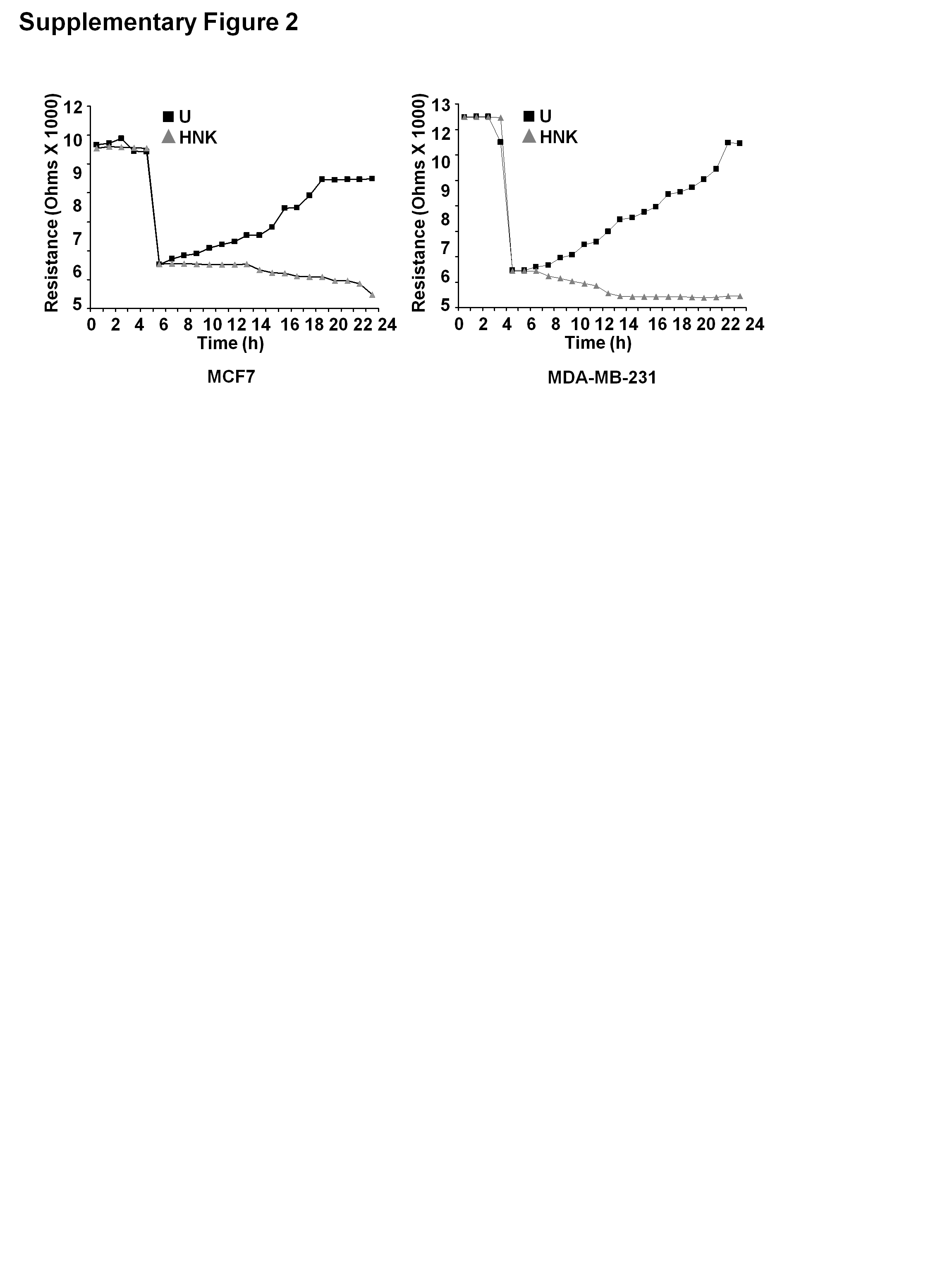

Supplement: Additional file 2 — Figure S2. Honokiol inhibits migration and invasion of breast cancer cells. Confluent layer of MCF7 and MDA-MB-231 breast cancer cells grown on electric cell-substrate impedance sensing (ECIS) 8W1E plates was subjected to an elevated voltage pulse of 40 kHz frequency, 3.5 V amplitude for 30 seconds to create a wound, and resistance was measured for 24 hours in the presence (HNK, 2.5 μM) and absence (U) of honokiol to monitor the migration of breast cancer cells. Honokiol treatment inhibited migration of breast cancer cells in an ECIS assay. All the experiments were performed thrice in triplicate. [file bcr3128-S2.TIFF]

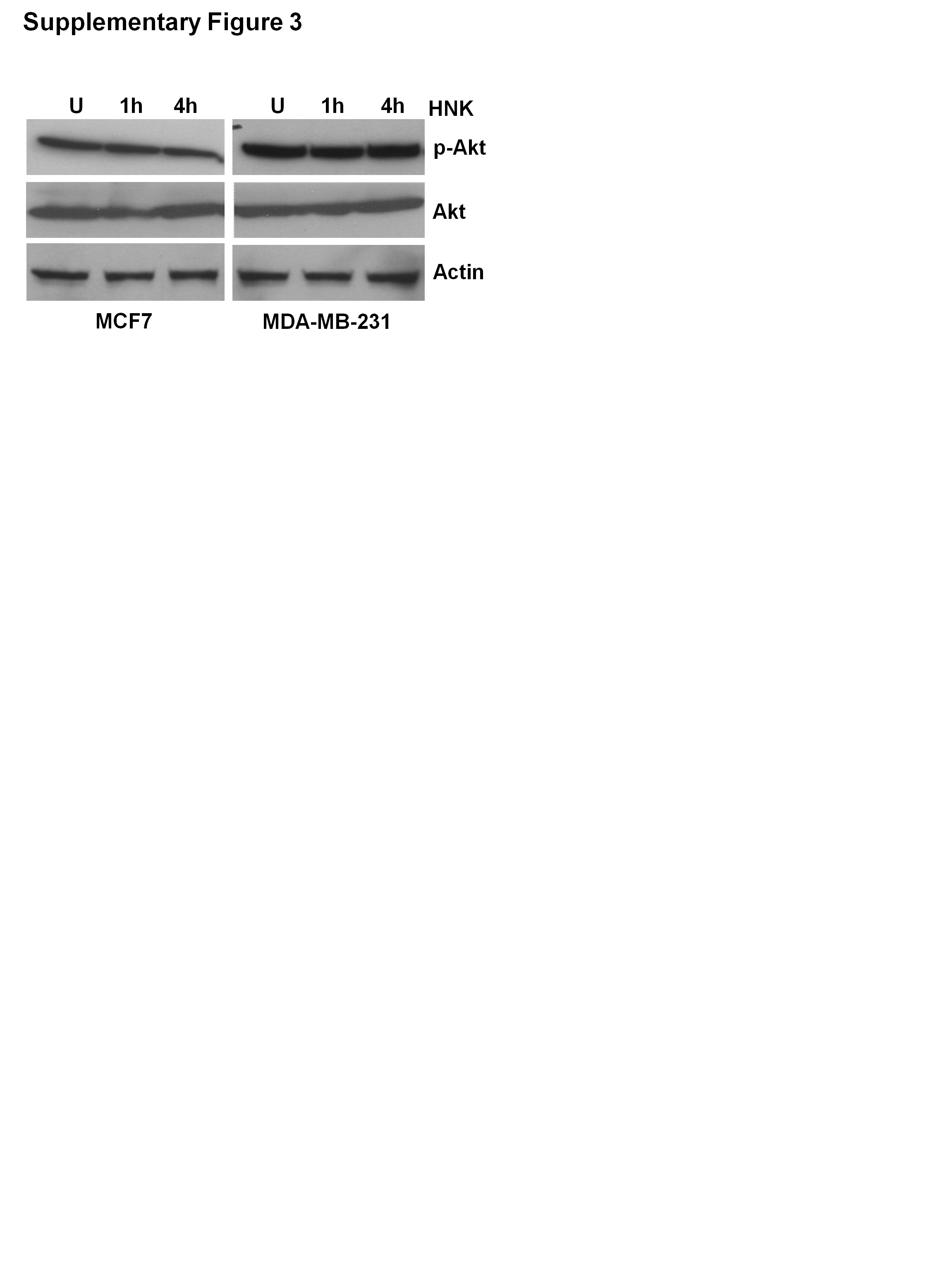

Supplement: Additional file 3 — Figure S3. Honokiol does not modulate Akt activation in breast cancer cells. MCF7 and MDA-MB-231 cells were treated with honokiol (HNK, 2.5 μM) for indicated time intervals. U, untreated cells. Total protein was isolated, and equal amounts of proteins were resolved with SDS-PAGE and subjected to immunoblot analysis by using specific antibodies for phosphorylated Akt. The membranes were reblotted by using total Akt antibodies as controls. The blots are representative of multiple independent experiments. [file bcr3128-S3.TIFF]

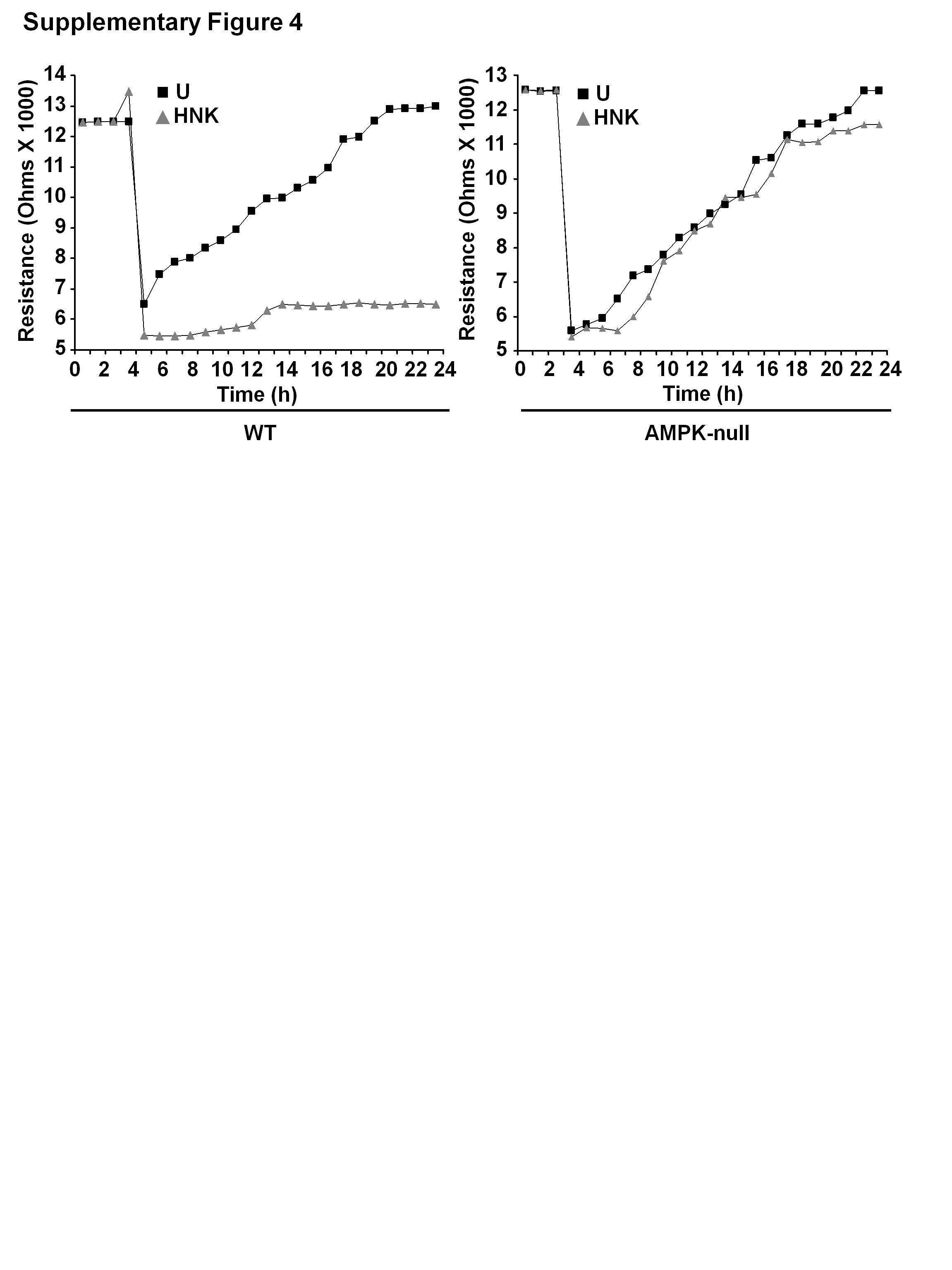

Supplement: Additional file 4 — Figure S4. AMPK knockdown abrogates honokiol-mediated inhibition of migration. Confluent layer of WT and AMPK-null MEFs grown on electric cell-substrate impedance sensing (ECIS) 8W1E plates was subjected to an elevated voltage pulse of 40 kHz frequency, 3.5 V amplitude, for 30 seconds to create a wound, and resistance was measured for 24 hours in the presence (HNK, 2.5 μM) and absence (U) of honokiol to monitor the migration of MEFs. All the experiments were performed thrice in triplicate. [file bcr3128-S4.TIFF]
